# Scar endometriosis, a form of abdominal wall endometriosis–a neglected obstetrical complication?

**DOI:** 10.1007/s00404-024-07834-2

**Published:** 2024-11-28

**Authors:** T. M. Gruber, K. Lange, G. S. Ebeling, W. Henrich, S. Mechsner

**Affiliations:** 1 Department of Obstetrics, Charité Universitätsmedizin Berlin, Berlin, Germany; 2 Endometriosis Center, Charité Universitätsmedizin Berlin, Berlin, Germany

**Keywords:** Abdominal wall endometriosis, Scar, Cicatrix, Post-operative complication

## Abstract

**Background:**

Scar endometriosis (EM) is defined by the presence of endometrial-like tissue outside the uterine cavity within the scar region after abdominal or pelvic surgery. It is a form of abdominal wall EM.

This systematic review addresses the question of whether women after cesarean delivery (CD) are more frequently affected by scar EM than women after other pelvic surgical procedures. The primary aim is to analyze the distribution of previous operations in patients with scar EM. Secondarily, symptoms, diagnosis, and treatment are described.

**Methods:**

A systematic literature search in MEDLINE (Pubmed) was performed. Twelve studies were included.

**Results:**

The terminology of scar EM is unspecific and the descriptions are, therefore, of limited comparability among authors. In 64–96%, patients with scar EM had a history of CD, followed by laparoscopy, laparotomy, and episiotomy. The main symptoms were pain, often cyclical, and the presence of local swelling. For diagnosis ultrasound, CT scan and MR imaging were used. All patients had undergone surgical resection and the diagnosis was confirmed.

**Conclusion:**

Most often scar EM develops after CD. Diagnosis and treatment are often delayed. As an objective classifications system is missing, we propose a simple objective descriptive tool for abdominal wall EM.

## Introduction

Endometriosis (EM) is a chronic inflammatory disease defined by the presence of endometrial-like tissue outside the uterine cavity [1]. Ectopic epithelial and stromal cells as well as smooth muscle cells manifest mainly in the pelvis. The most common sites are the peritoneum of the internal genitalia, the ovaries and the myometrium, then called adenomyosis (AM) [2]. Organs such as the liver, diaphragm, or lungs are less commonly affected [3]. The estimated current prevalence of EM is 10% of all women [4]. Typical symptoms include chronic lower abdominal pain, dysmenorrhoea, dyspareunia, dyschezia, dysuria, and unfulfilled desire to have children. A presumptive diagnosis can be made by medical history, palpation, and vaginal ultrasound [5]. EM is an estrogen-dependent disease and it can be treated hormonally with combined oral contraceptives, progestogen monotherapy, and the local progestogen-releasing intrauterine device (IUD). Pain associated with EM is treated early, at low doses and regularly, with non-steroidal analgesics according to the WHO regimen [6]. If hormone therapy is not sufficient, all visible EM lesions can be removed during surgery. Post-operative hormone therapy is again indicated [5]. The recurrence rate after surgery and endocrine therapy is reported up to 50–80% [5].

**Fig. 1:**
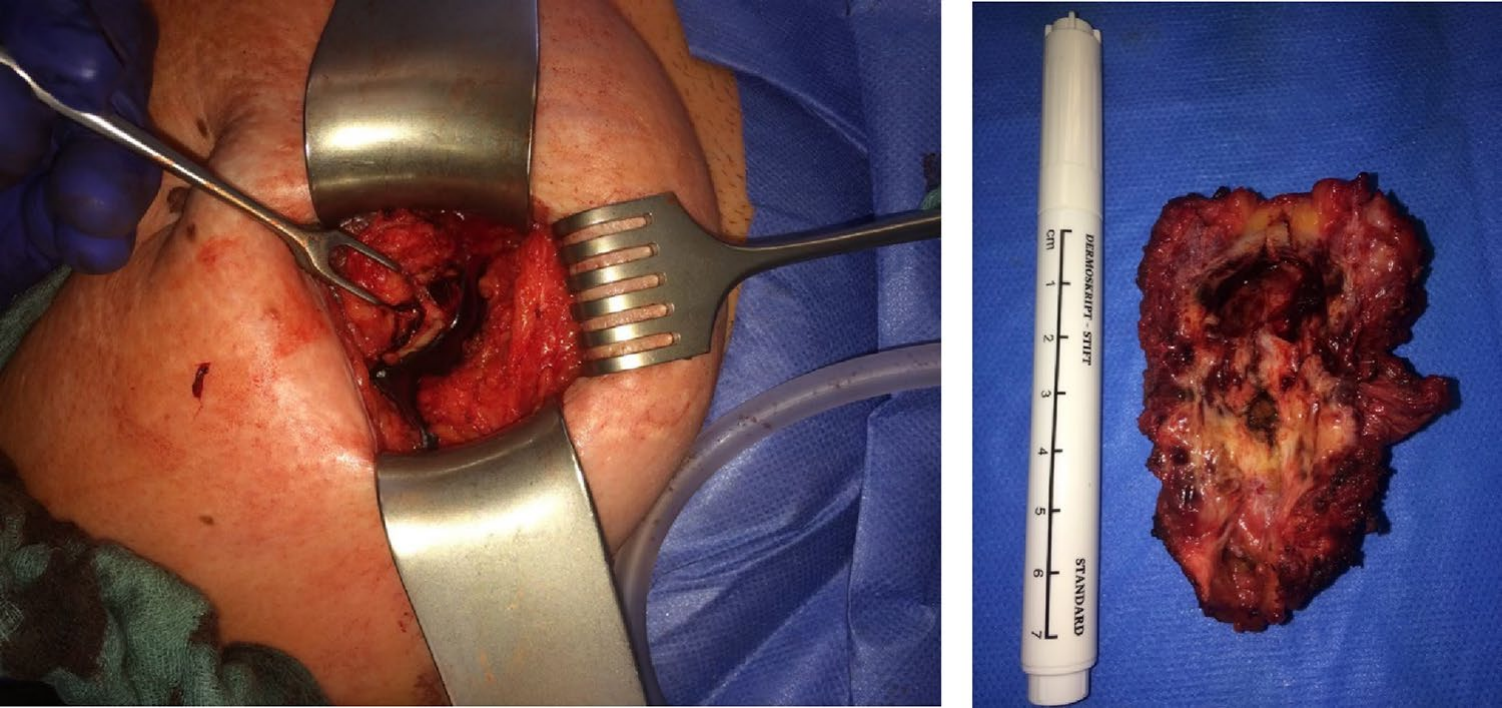
Intraoperative presentation of scar EM after surgery with a size of 7 cm, measured after local excision

Abdominal wall EM is a rare form of EM [2]. It is defined by the ectopic presence of endometrial-like tissue and smooth muscle cells in the subcutaneous fat layer and/or in the muscles of the abdominal wall [7, 8]. It can be diagnosed primarily without any previous surgery, for example as primary umbilical EM [8, 9]. More often, secondary abdominal wall EM occurs after previous surgery and is known as scar EM [10] (Fig. 1.).

The primary aim of the following review of literature is to analyze the distribution of previous surgical procedures among women with scar EM. Secondary aim is to summarize the symptoms, diagnosis, and treatment of abdominal wall EM.

Case reports and case series are the main types of publications on this condition. The overall scientific quality is poor due to the lack of prospective studies and the heterogeneous description of the disease [11].

## Methods

### Search strategy

A systematic literature search was conducted in MEDLINE (Pubmed) in the period from 1 to 13 January 2022 and again from 20 to 21 May 2024. A structured search strategy was developed using standardized medical subject headings (MeSH terms). The search in MEDLINE (Pubmed) was: “Endometriosis”[Mesh] AND “Cicatrix”[Mesh] AND “Cesarean Section”[Mesh]. A language filter was applied for English and German. There was no time limit, and articles from 1956 to 2024 were included. In an initial selection process, all results were eliminated if it was clear from the title that they did not fit the given research question. All potentially relevant references were obtained in full text and analyzed for compliance with the inclusion criteria. In addition, the bibliographies of the included studies were manually searched for studies not previously included. The selection process was documented in an Excel spreadsheet with the individual inclusion and exclusion criteria of all results in a comprehensible manner.

### Inclusion and exclusion criteria

All publications with a study population suffering from scar EM were included in the analysis. To be included, surgical procedures had to be recorded in the patient’s medical history. All articles that only included patients with scar EM after cesarean delivery (CD) were excluded, as the distribution of surgery among scar EM patients was the scope of the analysis.

### Quality assessment

The quality of all sources was assessed. The first step was to assess the methodology, and thus the inclusion criteria of the study population. Histopathological confirmation of the diagnosis in all patients was defined as a study quality characteristic. The publications were then reviewed for consistency of data. In particular, figures in the text and tables were compared. After careful analysis of the available full texts, all studies with ≤ 5 patients were excluded. This was due to the lack of power of case reports. Those mostly reflected exceptional individual cases of scar EM.

### Data collection

The characteristics of the study population were summarized. Those included age, previous surgery, symptoms and latency from last surgery to onset of symptoms, duration of symptoms, diagnosis, treatment, size and location of EM focus, histology, follow-up, and recurrence. After initial data collection, the extracted data were re-checked for completeness and accuracy. All results were also expressed as a percentage of the study population to ensure comparability between studies.

## Results

**Fig. 2:**
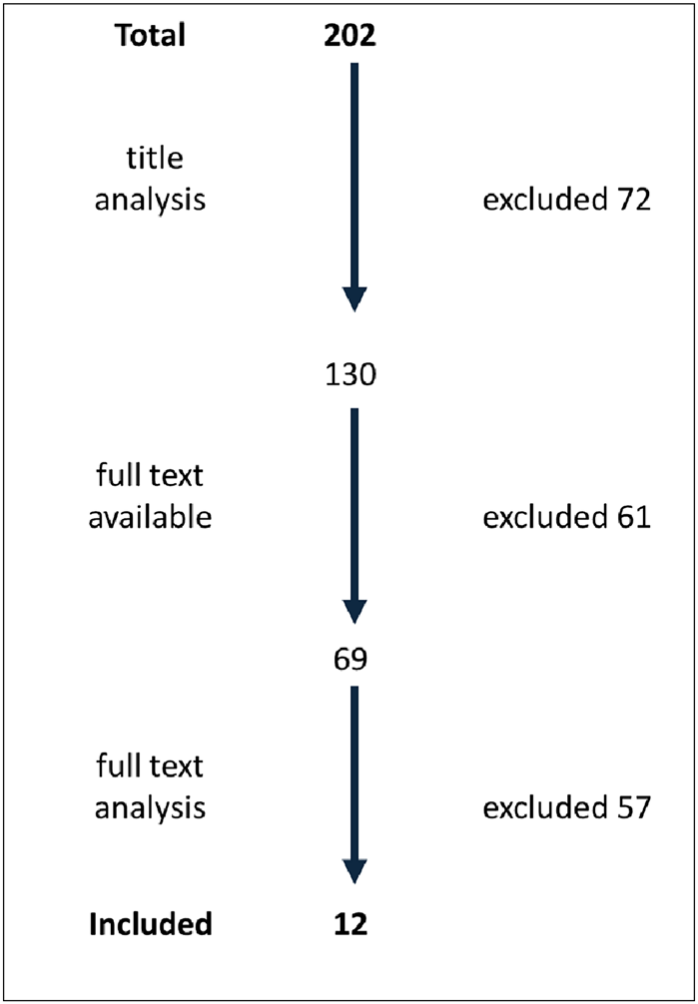
Flowchart of selection process of scientific literature for the review

The PubMed search yielded 198 hits. In addition, four articles were found by manual search in bibliographies. Based on the title, 72 of 202 sources were excluded. Of the remaining 130 results, 61 sources could be obtained neither via the license agreements of the Charité Medical Library nor via an advanced online search. A further 57 studies were excluded based on the inclusion and exclusion criteria. The analysis finally included 12 English-language studies (Fig. 2).

### Study characteristics

All included studies retrospectively analyzed patients with scar EM and examined them for various parameters.

The smallest study included 6 patients [12] and the largest study 83 patients [13]. The period of data inclusion was between 3 [14] and 26 years [15]. In the study with the youngest patients, the average age was 29 years [14] and in the one with the oldest patients, it was 35 years [16].

### Quality of the included studies

Our primary inclusion criterion was the histopathological confirmation of scar EM diagnosis and we only included studies that met this criterion. Second, we intended to present data on the history of EM and AM of patients with scar EM. Information on the patient’s history of EM was missing in 6 out of 12 studies, and no study provided information on the patient’s history of AM. Moreover, reporting of recurrences and follow-up among patients varied greatly and decreases the comparability across the selected studies. With this critique in mind, we still included as many articles as possible due to the limited availability of studies on the topic of interest.

### Surgery prior to scar EM

The distribution of previous surgical interventions was heterogeneous (see Table 1). The proportion of CD ranged from 64% [15] to 96% [17]. Moreover, patients with primary abdominal wall EM without prior surgery were described in three studies with localization of the EM site mostly umbilical but also in the groin [13, 15, 18].

**Table 1:** Surgical procedures in patients with scar EM prior to diagnosis

Author	Year	Population	CD in %	LSK in %	Laparotomy in %	Episiotomy in %
Benedetto et al. [13]	2022	83	66	10	n/a	n/a
Tatli et al. [21]	2018	14	86	n/a	n/a	n/a
Khan et al. [16]	2017	34	88	47	94	n/a
Vellido-Cotelo et al. [19]	2015	17	82	6	n/a	12
Ecker et al. [18]	2014	65	82	43	20	n/a
Goel et al. [12]	2011	6	83	n/a	n/a	17
Akbulut et al. [20]	2010	15	87	13	n/a	n/a
Nominato et al. [15]	2010	72	64	n/a	n/a	26
Bektas et al. [22]	2010	40	90	n/a	n/a	n/a
Leite et al. [23]	2009	33	94	n/a	n/a	6
Teng et al. [17]	2008	22	96	n/a	n/a	n/a
Gunes et al. [14]	2005	11	82	n/a	n/a	9

### Symptoms of women with scar EM

Patients in all studies suffered most frequently from pain and palpable EM lesions (see Table 2). Women reported pain in 41% [19] up to 100% [14, 16] of the cases often described as cyclical [13, 14, 20, 21]. Palpable EM lesions were also a leading symptom, whereas bleeding of the nodule was only described in three studies [12, 22, 23].

**Table 2:** Symptoms of patients described

Author	Year	Population	Pain (cycl./noncycl.) (%)	Palp. EM lesion (%)	Concomitant pelvic EM (%)	Dysmenorrhea (%)	Dyspareunia (%)	Bleeding (%)
Benedetto et al. [13]	2022	83	72 (100/0)	33	n/a	n/a	n/a	n/a
Tatli et al. [21]	2018	14	71 (100/0)	100	0	n/a	n/a	n/a
Khan et al. [16]	2017	34	100 (n/a)	n/a	0	12	n/a	n/a
Vellido-Cotelo et al. [19]	2015	17	41 (40/60)	82	14	21	n/a	n/a
Ecker et al. [18]	2014	65	74 (n/a)	63	n/a	17	11	n/a
Goel et al. [12]	2011	6	100 (67/33)	100	0*	n/a	n/a	17
Akbulut et al. [20]	2010	15	87 (85/15)	73	20	n/a	n/a	n/a
Nominato et al. [15]	2010	72	70 (64/36)	79	n/a	3	6	n/a
Bektas et al. [22]	2010	40	85 (47/53)	100	3	3	3	3
Leite et al. [23]	2009	33	94 (77/23)	91	n/a	n/a	n/a	3
Teng et al. [17]	2008	22	91 (100/0)	91	n/a	n/a	n/a	n/a
Gunes et al. [14]	2005	11	100 (100/0)	100	0	n/a	9	n/a

### Time to onset of symptoms

The reported time from surgery to the onset of symptoms was in average 11 months [20] to 60 months [14]. The largest range was 2–240 months [15].

### Diagnostic procedures

The main tools used for diagnosis of scar EM were ultrasound, followed by CT and MRI scans (see Table 3). Pre-operative fine needle aspiration was used rarely and only confirmed the diagnosis of EM in 50% according to one study [22]. In two studies, only a clinical examination was performed [16, 19].

**Table 3:** Diagnostic tools used in patients with scar EM

Author	Year	Population	US	CT	MRI	FNA
Benedetto et al. [13]	2022	83	78,3	n/a	21,7	n/a
Tatli et al. [21]	2018	14	50	43	7	n/a
Khan et al. [16]	2017	34	24	21	15	n/a
Vellido-Cotelo et al. [19]	2015	17	35	6	6	52
Ecker et al. [18]	2014	65	34	40	25	3
Goel et al. [12]	2011	6	17	n/a	n/a	67
Bektas et al. [22]	2010	40	88	40	25	20
Gunes et al. [14]	2005	11	91	n/a	n/a	n/a

### Management of abdominal wall EM

All patients underwent surgical resection of EM lesions. In big lesions, a mesh was inserted intraoperatively to repair a defect caused by the resection. The proportion varied between 3% [23] and 40% [20]. When measured, the mean size of the lesions was 30 mm within a range reported between 3 and 90 mm [15, 24].

All studies described the location of the EM nodule. Most publications stated a direct relationship between the EM lesion and scar. In one study, only 47% of the removed endometrial-like tissue was found to be directly related to the scar [19]. In contrast, in four studies, 100% of the tissue to be removed was present in or near the scar [12, 14, 17, 20, 23].

One study reported preoperative and two publications reported post-operative drug therapy. Goel et al. treated two patients preoperatively with danazol (33%; *n* = 2/6) as a primary treatment option. As the effect was not sufficient, both patients subsequently underwent surgery [12]. In a second study, all patients were postoperatively prescribed hormonal therapy: of those, 1 patient was prescribed danazol (7%), 1 buserelin (7%), and 13 patients were prescribed oral contraceptives (87%), of which 3 patients were prescribed buserelin in combination with oral contraceptives (20%) (*n* = 15) [20].

### Concomitant EM

Six out of twelve studies evaluated the amount of concomitant pelvic EM in patients with abdominal wall EM. Two studies found that no woman had associated pelvic EM [14, 16]. The highest percentage of patients with concomitant EM was 20% [20]. The mean percentage of concomitant EM was 5.8%. No study evaluated whether AM was present.

### Follow-up after treatment of scar EM

Three studies reported an average follow-up of 25 months [14, 20, 21]. In two other studies, the follow-up took place after 6–150 months and 9–144 months [12, 17]. Only one study reported a drop-out rate of 18% (*n* = 7/40) [22]. No recurrences were recorded in four studies [12, 14, 17, 21]. The recurrence rates reported ranged from 0 to 13% (see Table 4).

**Table 4:** Follow-up and recurrences in patients with scar EM

Author	Year	Population	Follow-up	recurrence
Benedetto et al	2022	83	Time to diagnosis 5.2 years	n/a
Tatli et al	2018	14	About 9 months	0
Khan et al. [16]	2017	34	36–65 months	6
Vellido-Cotelo et al. [19]	2015	17	n/a	6
Ecker et al. [18]	2014	65	n/a	n/a
Goel et al. [12]	2011	6	9–144 months	0
Akbulut et al. [20]	2010	15	22 ± 14 months (5–47)	13
Nominato et al. [15]	2010	72	n/a	1
Bektas et al. [22]	2010	40	Yes, period not defined	9
Leite et al. [23]	2009	33	n/a	6
Teng et al. [17]	2008	22	6–150 months	0
Gunes et al. [14]	2005	11	22 months (mean)	0

## Discussion

Scar EM is a long-term complication following surgical interventions. In our review, we analyzed studies that included women with scar EM after a variety of abdominal and pelvic interventions. The operation that bears the highest risk factor for women to develop scar EM is a CD. In 64% to 96%, CD was the surgery that preceded scar EM. The time interval between surgery and disease onset varies greatly. Main symptoms across studies were local, often cyclic pain and swelling. Concomitant pelvic EM was rarely studied. The rates of concomitant EM described by the authors ranged from 5.8% to 20% and adenomyosis was not evaluated. The diagnostic tool of choice was ultrasound followed by CT scan and MRI. All patients underwent surgical excision. Follow-up hormonal treatment is not standard across the studies and was rarely reported. As the follow-up and drop-out rates were not identical across the studies and missing in many cases, recurrence rates may only be a rough estimation. The authors report rates from 0 to 13%.

### Pathophysiology

The iatrogenic transplantation theory states that endometrium is transferred into the wound tissue during surgical procedures [25]. This applies in particular to obstetric and gynecological operations in which the uterus is opened [2, 10]. The estimated incidence in literature is often cited as 0.03% to 1.08% of all women after pelvic surgery, while the initial work this number is based on is a case series with 17 patients collected within 5 years in 1980 [20, 26].

Scar EM is probably triggered less frequently by minimally invasive procedures such as laparoscopy and more frequently by major surgery. A CD is always associated with close contact between the endometrium and wound tissue. Consequently, it can be assumed that endometrial cells are increasingly transferred to the surrounding area [27]. For this reason, Zhang et al. recommend thorough intraoperative irrigation the use of separate needles for uterine and abdominal closure and no use of sponges to clean the uterine cavity for CD [28].

We assume that the mechanical translocation of EM tissue is causative for the EM implantation within the scar tissue. In consequence, the amount of tissue affected by EM should also be considered as a risk factor for the development of scar EM. Therefore, in women with EM, who often undergo several surgical procedures, we assume a higher risk of developing scar EM than in the general population [29].

### Diagnosis and treatment

Due to the rarity of the disease, a false diagnosis (hernia, hematoma, lipoma) is often made preoperatively [16, 22]. If scar EM is suspected, a detailed medical history with a particular focus on previous operations and symptoms, a targeted physical examination, and a transabdominal ultrasound are performed for diagnostic purposes. If the diagnosis is unclear, MRI, CT and fine needle aspiration may also be used [2].

The terminology of abdominal wall EM varies and there is no classification system in place yet [30]. In the non-invasive and surgical description system #ENZIAN, scar EM as a form of abdominal wall EM can be noted under the FO, “o” for other EM localizations [31]. Detailed data are lacking.

The EM lesion usually appears well-defined, heterogeneous, hypoechogenic with internal vascularization [32]. To allow a precise description, we propose a new classification for abdominal wall EM including scar EM (Table 5). We suppose that within the classification, all forms of abdominal wall EM, primary and secondary, can be objectively described. We suggest a description of the lesions as, e. g., *secondary scar EM after CD, 2 a, c*.

**Table 5:** Classification for abdominal wall EM

Abdominal wall EM Classification	Umbilical	Scar
Primary/secondary		
Secondary (CD, Laps/Lap)		
Size		
1) < 1 cm		
2) 1–3 cm		
3) > 3 cm		
Symptoms		
a) Cyclical pain		
b) Cyclical swelling		
c) Cyclical bleeding		

The only curative treatment is resection of the affected tissue [33]. Histopathologically, the diagnosis is confirmed by finding endometrial glands, stroma or hemosiderin pigment [10]. Nevertheless, the indication to surgery is an individual process depending on the patient’s situation including symptoms, size of the lesions, family planning and personal circumstances. Primary hormone therapy can be a treatment option.

### Concomitant EM

The mean percentage of concomitant EM was 5.8% which is both less than the 13% previously described in scar EM patients [34] and less than the prevalence in the general population of about 10% [4]. Especially umbilical EM is associated with pelvic EM, whereas adenomyosis is linked to more extensive forms of scar EM after CD [35]. None of the reviewed studies evaluated whether there is an association between AM and scar EM. As the studies did not focus on concomitant EM, most of them evaluated its presence using neither standardized classification systems nor standardized examinations. We, therefore, assume that the percentage of concomitant EM in patients with EM is higher in the study collective. And vice versa, we postulate that in the cohort of women with EM, the risk of scar EM after surgery is also higher than in the general population.

### Limitations

There is a confounding effect by the existence of primary abdominal wall EM in up to 20% of the reported cases [13, 15, 18]. Moreover, the studies included women who had undergone more than one operation prior to the diagnosis of scar EM.

A detailed classification system for scar EM is lacking so that the comparison of the lesions across the study populations is difficult. At the same time, the terminology itself is inconsistent. Many different terms are used for the disease complex: abdominal wall EM, scar EM, cicatricial EM, abdominal incision EM, cutaneous EM and others. In terms of methodology, the publications differed in their inclusion criteria (symptoms, diagnosis, resection, histopathology of scar and/or abdominal wall EM).

## Conclusion

Scar EM is a post-operative complication that is lacking attention. The incidence in the general population with a maximum of 1% is underestimated in the population at risk, namely women with EM/AM. To add an objective descriptive tool to the diagnostic algorithm, we suggest a new classification system for abdominal wall EM. Prospective research is needed to evaluate the risk of scar EM in women with EM/AM. Moreover, clinicians need to consider this post-operative complication during the process of shared decision-making while planning an operation.

## Data Availability

Not applicable.
